# Effects of epidermal growth factor receptor (EGFR) tyrosine kinase inhibitors (TKIs) targeted combined chemotherapy on immune function, tumor markers and oxidative stress in patients with stage IV lung adenocarcinoma

**DOI:** 10.12669/pjms.39.3.7397

**Published:** 2023

**Authors:** Yanxiao Kun, Jian Wu, Shaomu Chen

**Affiliations:** 1 Yanxiao Kun, Department of Surgery II, Suzhou Hospital of Integrated, Traditional Chinese and Western Medicine, Suzhou 215000, Jiangsu Province, P.R. China; 2 Jian Wu, Department of Surgery II, Suzhou Hospital of Integrated, Traditional Chinese and Western Medicine, Suzhou 215000, Jiangsu Province, P.R. China; 3 Shaomu Chen, Department of Thoracic Surgery, The First Affiliated Hospital of Soochow University, Suzhou 251000, Jiangsu Province, P.R. China

**Keywords:** Epidermal growth factor receptor, Tyrosine kinase inhibitor, Targeting, Stage-IV lung adenocarcinoma, Immunity, Tumor markers, Oxidative stress

## Abstract

**Objective:**

To investigate the effects of EGFR-TKI combined chemotherapy on immune function, tumor markers and oxidative stress in patients with stage-IV lung adenocarcinoma.

**Methods:**

This retrospective observational study included 116 patients with stage-IV lung adenocarcinoma, treated in The First Affiliated Hospital of Soochow University from January 2021 to January 2022. According to the treatment records, 60 patients that received pemetrexed + cisplatin for four courses were set as a Control-group and 56 patients that received EGFR-TKI + pemetrexed + cisplatin for four courses were set as an Observation-group. Changes in immune function, tumor marker levels and oxidative stress level in the two groups were analyzed and compared.

**Results:**

After the treatment, levels of CD3^+^, CD4^+^, IgG and IgM in the Control-group were significantly lower than those before the treatment. EGFR-TKI + pemetrexed + cisplatin resulted in levels of CD3^+^, CD4^+^, IgG and IgM higher than before the treatment, and compared to the Control-group (*p*<0.001). After the treatment, the levels of NSE, serum CEA, serum CA125, CYFEA21-1 in both groups were significantly lower than those before treatment, and lower in the Observation-group (*p*<0.001). After the treatment, VEGF and MMP9 levels in both groups were significantly lower than those before treatment, and markedly lower in the Observation-group (*p*<0.001).

**Conclusion:**

Compared with systemic chemotherapy, EGFR-TKI targeted combined chemotherapy for stage-IV lung adenocarcinoma is associated with enhanced immune function of patients. It more effectively inhibits the growth and proliferation of tumor cells and reduces the level of oxidative stress.

## INTRODUCTION

Lung cancer is a common malignant tumor that can be divided into two types: small cell lung cancer and non-small cell lung cancer. The latter accounts for more than 80% of all diagnosed lung cancer cases.^1^ Non-small cell lung cancer (NSCLC) includes adenocarcinoma and squamous cell carcinoma.^2^,^3^ According to the accepted staging system, NSCLC may be graded as stage I (21%), stage II (4%), stage III (26%), and stage IV (40%), with the median overall survival of stage IV patients ranging from 7 to 12.2 months.^4^,^5^ Most lung adenocarcinomas originate from bronchial mucosal epithelium, and are often asymptomatic, especially in the early stage. The tumors originating in major bronchus are diagnosed earlier, while those originating in the peripheral bronchus are diagnosed late.^6^ Therefore, most of the patients are diagnosed in stage-IV, and their survival could only be prolonged by radiotherapy, chemotherapy and targeted therapy.^7^ However, these approaches have limitations, since radiotherapy and chemotherapy may cause undifferentiated cell destruction, which seriously affects the quality of life of patients in the later stages of the disease.^8^

As such there is an urgent need to develop alternative treatment methods to improve quality of life of lung adenocarcinoma patients.^9^ In recent years, with the development and application of molecular biology technology in oncology, targeted drugs have gradually become one of the research hotspots of precision tumor therapy, as they are associated with lower toxicity and less side effects.^10^ In recent years, targeted therapies have gradually become among of the most important means to treat advanced non-small cell lung cancer.^11^ Among them, epidermal growth factor receptor (EGFR) – tyrosine kinase inhibitor (TKI) is considered the most promising drug that can effectively improve the survival rate of patients with advanced lung cancer.^9^,^12^ However, although EGFR-TKI targeted therapy can effectively improve clinical symptoms of lung adenocarcinoma patients, it is inevitable that acquired drug resistance will eventually appear. If the treatment is interrupted after the occurrence of acquired drug resistance, it may lead to rapid tumor progression. Therefore, it is necessary to combine EGFR-TKI targeted therapy with other therapeutic drugs.

Pemetrexed has been reported to target thymidylate synthase, and study also reported that EGFR-TKI can down-regulate TS expression.^13^,^14^ La Monica et al. also reported that gefitinib combined with pemetrexed prevented the acquisition of TKI resistance in NSCLC cell lines carrying EGFR-activating mutation.^15^ The synergistic effect of TKI and pemetrexed has great promise in lung adenocarcinoma treatment. At present, studies have investigated the efficacy of EGFR-TKI combined with chemotherapy from the perspective of overall survival, but there are few reports comparing EGFR-TKI targeted combined chemotherapy with EGFR-TKI or chemotherapy alone in terms of immune function, tumor markers and oxidative stress in stage-IV lung adenocarcinoma.^16^-^18^ Therefore, this study mainly discussed the effects of EGFR-TKI targeted combined chemotherapy on immune function, tumor markers and oxidative stress level of stage-IV lung adenocarcinoma patients.

## METHODS

A total of 139 patients with stage-IV lung adenocarcinoma, treated in The First Affiliated Hospital of Soochow University from January 2021 to January 2022 were selected for this retrospective observational study. All patients were screened for eligibility and 23 patients who did not meet the inclusion and exclusion criteria were excluded. The patients were grouped retrospectively according to the treatment mode: patients that received pemetrexed + cisplatin were set as the Control-group (n=60), and patients that received EGFR-TKI + pemetrexed + cisplatin were set as the Observation-group (n=56).

### Inclusion criteria:

- Patients met the clinical diagnostic criteria for lung adenocarcinoma and TNM stage was stage-IV;^19^,^20^
- All received chemotherapy for the first time;
- EGFR mutation was confirmed by genetic testing;
- The Karnofsky performance status (KPS) score ≥70 points;^21^
- The survival period was more than three months;^22^
- Complete clinical data.

### Exclusion criteria:

- Patients with concomitant systemic disorders like interstitial pneumonia or primary malignancy;
- Serious dysfunction of heart, liver and kidneys;
- Allergic to pemetrexed, cisplatin and gefitinib;
- Patients with cognitive and verbal communication impairments;

### Ethical approval:

This study was approved by the medical ethics committee of our college (No.: 2022-594; Date: 2022-03-01).

### Systemic chemotherapy:

On the first day, pemetrexed 500 mg/m^2^ and 100 ml normal saline were used for intravenous drip. Cisplatin 40 mg/m^2^ and 500 ml normal saline were injected intravenously for three days. The first administration time of cisplatin was 30 minutes after pemetrexed intravenous drip. Each course of treatment lasted for three weeks. After three weeks of treatment, patients rested for one week, and the treatment was continued for a total of four courses.

### EGFR-TKI targeted therapy:

On the second to 14th days after the start of systemic chemotherapy with cisplatin (as described above), gefitinib was administered orally, 250 mg/ time, once a day, three weeks for a course of the treatment. After three weeks of treatment, patients rested for one week, and the treatment was continued to for a total of four courses. TKI treatment was stopped after the end of chemotherapy.

Immune function index, tumor marker index levels and oxidative stress index levels of patients were collected before and after the treatment. Briefly, 3ml of abdominal peripheral venous blood was collected in the morning, and separated using 5417R bench top high-speed freezing centrifuge (Eppendorf, Germany) at 100rpm/minute for 15 minutes. Levels of CD3^+^ and CD4^+^ in the serum were measured by flow cytometry using Epicsxl flow cytometer (Beckman Kurt, United States). BS-380 automatic biochemical analyzer (Mindray Medical International Co., Ltd) was used to detect the levels of serum IgG and IgM, Specific kits were purchased from Shenzhen Jingmei Biotechnology Co., Ltd; The levels of neuron specific enolase (NSE), serum carcinoembryonic antigen (CEA), serum carbohydrate antigen (CA125), cytokeratin-19 fragment antigen 21-1(CYFEA21-1), vascular endothelial growth factor (VEGF) and matrix metalloproteinase-9 (MMP9) were detected by chemiluminescence.

### Statistical analysis:

Sample size was calculated using R package (Version 4.0.2, RStudio Inc., USA). A calculated sample of 53 patients was required in each group to detect an effect size of 0.55 between the two groups with 80% power and P-value < 0.05. It was done using SPSS 22.0 software. The counting data was expressed in n (%) and analyzed by *χ^2^* inspection. The measurement data is represented by (*χ̅*±*S*), and *t*-test was performed. *p*<0.05 indicated statistical significance.

## RESULTS

The average age of the patients was 64.11±6.86 years, the average weight was 64.37±9.35kg, and the average course of the disease was 3.22±1.21 years. There was no significant difference in the basic data between the two groups, as shown in Table-I (p*>*0.05).

**Table-I:** Comparison of basic clinical features between the two groups[n(%),*χ̅*±*S*].

Group	Gender (Male/Female)	Mean age (years)	Mean weight (kg)	Mean course of disease (years)	Smoking status (Yes/No)	KPS Score
Control-group (n=60)	25/35	64.41±6.70	63.83±9.12	3.33±1.20	34/26	74.83±6.24
Observation-group (n=56)	21/35	63.78±7.08	64.96±9.03	3.11±1.22	36/20	75.89±7.08
χ2/t	0.210	0.493	-0.670	1.007	0.703	-0.856
p-Value	0.647	0.623	0.504	0.316	0.402	0.394

As summarized in Table-II, there was no significant difference in the immune function between the two groups before the treatment (*p*>0.05). Treatment with the systemic chemotherapy (Control-group) was associated with significant decrease in the levels of CD3^+^, CD4^+^, IgG and IgM compared to before treatment. In contrast, CD3^+^, CD4^+^, IgG and IgM levels in the Observation-group were significantly higher than those before treatment, higher than those in the Control-group (*p*<0.001).

**Table-II:** Comparison of immune function between the two groups (*χ̅*±*S*).

Group	CD3+ (%)		CD4+ (%)		IgG (g/L)		IgM (g/L)	

	Before treatment	After treatment	Before treatment	After treatment	Before treatment	After treatment	Before treatment	After treatment
Control-group (n=60)	57.56±4.60	48.46±3.75*	42.75±3.49	31.65±3.22*	16.79±2.23	10.98±2.34*	1.94±0.20	1.54±0.17*
Observation-group (n=56)	56.80±4.90	59.03±5.11*	43.34±3.89	46.30±3.50*	16.50±2.35	18.51±2.62*	1.98±0.22	2.30±0.28*
t	0.864	-12.618	-0.859	-15.460	0.681	-16.329	-0.861	-17.461
p-Value	0.389	<0.001	0.392	<0.001	0.497	<0.001	0.391	<0.001

***Note:***

* indicates the comparison within this group with that before treatment, p<0.001.

Before the treatment, there was no significant difference in the level of tumor markers between the two groups (*p*>0.05). After treatment, the levels of neuron specific enolase (NSE), serum carcinoembryonic antigen (CEA), serum carbohydrate antigen (CA125), and cytokeratin-19 fragment antigen 21-1(CYFEA21-1) in both groups decreased significantly, and this decrease was significantly lower levels in the Observation-group (*p*<0.001), as shown in Table-III. Before the treatment, there was no significant difference in the level of oxidative stress between the two groups (*p*>0.05). After treatment, the levels of vascular endothelial growth factor (VEGF) and matrix metalloproteinase-9 (MMP9) in the two groups were significantly lower than those in the Control-group (*p*<0.001), as shown in Table-IV.

**Table-III:** Comparison of Tumor marker levels between the two groups (*χ̅*±*S*).

Group	NSE (ng/mL)		CEA (ng/mL)		CA125 (U/m L)		CYF―1 (ng/mL)	

	Before treatment	After treatment	Before treatment	After treatment	Before treatment	After treatment	Before treatment	After treatment
Control-group(n=60)	30.03±4.27	20.95±3.90*	41.08±4.40	29.98±4.07*	91.05±5.17	55.00±4.85*	8.97±1.52	6.82±1.37*
Observation-group (n=56)	30.96±4.55	17.09±4.24*	40.60±4.21	25.39±3.78*	91.44±5.45	37.33±5.01*	8.90±1.73	4.86±1.53*
t	-1.136	5.098	0.594	6.276	-0.402	19.274	0.230	7.262
p-Value	0.258	<0.001	0.554	<0.001	0.689	<0.001	0.819	<0.001

***Note:***

* indicates the comparison within this group with that before treatment, p<0.001.

**Table-IV:** Comparison of oxidative stress levels between the two groups (*χ̅*±*S*).

Group	VEGF (ng/mL)		MMP9 (ng/mL)	

	Before treatment	After treatment	Before treatment	After treatment
Control-group (n=60)	453.23±50.37	303.06±47.21*	1863.93±202.14	1468.03±196.13*
Observation-group (n=56)	443.46±49.41	236.25±42.11*	1853.00±175.83	1141.92±172.32*
t	1.053	8.023	0.310	9.485
p-Value	0.294	<0.001	0.757	<0.001

***Note:***

* indicates the comparison within this group with that before treatment, p<0.001.

## DISCUSSION

This study analyzed the effects of EGFR-TKI targeted combined chemotherapy on immune function, tumor markers and oxidative stress level in patients with stage-IV lung adenocarcinoma. The results showed that EGFR-TKI targeted combined chemotherapy could enhance the immune function of patients compared with systemic chemotherapy alone. We may speculate that IgG and IgM participate in humoral immunity, CD4^+^ is involved in regulating or assisting immune response, CD8^+^ can inhibit and kill T-lymphocytes.^23^-^25^ EGFR-TKI targeted therapy only mildly affects the reproduction of normal tissue cells, and will not cause damage to normal tissues. Therefore, it has a small impact on the immune function of the body. Combined with chemotherapy, it can play a synergistic role, improve the immune function of patients and promote the anti-tumor effect.^26^,^27^

The results of this study also show that EGFR-TKI targeted combined chemotherapy is associated with improved levels of tumor markers and improved treatment effect. Xu A et al^28^ showed that EGFR-TKI targeted therapy applied to patients with advanced EGFR mutant NSCLC can downregulate the levels of CEA, CYFRA21-1 and MMP-9, effectively inhibit angiogenesis and enhance the immune function of patients. Li X et al^29^ compared Gefitinib and Osomertinib in the treatment of NSCLC patients, and the results showed that both drugs had good effects. Our results are consistent with the results of the above studies. EGFR-TKI is a small molecule drug that can act on the receptor tyrosine kinase region in the cells, effectively inhibit tyrosine kinase phosphorylation and downstream signal transduction, and promote cancer cell apoptosis, anti-angiogenesis, anti-differentiation and proliferation, and anti-cell migration. Thus, by inhibiting the tyrosine kinase activity required in the process of tumor cell differentiation and metastasis, EGFR-TKI targeted therapy is able achieve the effect of “killing” tumor cells.^30^,^31^ Therefore, gefitinib, as a EGFR-TKI, inhibits kinase activity by competitively interacting with the ATP-binding site of EGFR, thus preventing auto-phosphorylation and thereby blocking EGFR-induced activation of downstream signaling, which leads to apoptosis in cells with EGFR mutations. Padda SK et al^32^ pointed out that EGFR-TKI targeted therapy can effectively improve lung ventilation and reduce the level of oxidative stress in patients.

The results of our study showed that after the treatment, VEGF and MMP-9 levels in the Observation-group were significantly lower than those before treatment and lower than those in the Control-group (*p*<0.001), indicating that in agreement with previous results, EGFR-TKI targeted combined chemotherapy can effectively improve the level of oxidative stress and improve the prognosis of patients. It is possible that EGFR-TKI targeted therapy can effectively block the transmission of EGFR-related energy information, inhibit tumor cells from proliferating, and promote their apoptosis. At the same time, the treatment will significantly reduce levels of protein synthesis and secretion, resulting in a decrease in the growth rate of vascular endothelial cells.

This in turn can effectively improve patients’ lung ventilation, reduce the level of oxidative stress, and improve the prognosis.^33^ In addition, Hao Z et al^34^ showed that the incidence of adverse reactions after NLCSC chemotherapy was 28.3% (34/120), and the 1-year, 2-years and 3-years survival rates were 38.3%, 15.0% and 10.0%, respectively. However, this study did not observe the occurrence of adverse reactions and survival rate, which is also the focus of follow-up research.

In this study, we only investigated the effect of Gefitinib + pemetrexed + cisplatin, it is clinically important to further investigate how the sensitivity to clinical chemotherapeutic agents changes after the development of acquired resistance to TKI therapy in patients with advanced disease and to identify the most optimal combination treatment protocol in the future.

### Limitations:

This study has some limitations. This is a retrospective study with a relatively small sample size and short observation time. This may lead to a certain bias in the judgment of curative effect. Further studies with larger sample size are needed to confirm our findings and to explore the long-term survival of lung adenocarcinoma patients after EGFR-TKI targeted therapy. Additionally, since our study was focused on the effects of EGFR-TKI combined chemotherapy on immune function, tumor markers and oxidative stress, it did not address the effect of the treatment on the incidence of adverse reactions and overall survival rate.

## CONCLUSION

Compared with systemic chemotherapy, EGFR-TKI targeted combined chemotherapy for stage-IV lung adenocarcinoma can increase the immune function of patients, more effectively inhibit the growth and proliferation of tumor cells, and reduce the level of oxidative stress.

### Authors’ contributions:

**YK:** conceived and designed the study.

**JW** and **SC:** collected the data and performed the analysis.

**YK:** was involved in the writing of the manuscript and is responsible for the integrity of the study.

All authors have read and approved the final manuscript.
